# Durvalumab induced immune-related agranulocytosis after conversion surgery in a patient with intrahepatic cholangiocarcinoma: a case report

**DOI:** 10.3389/fimmu.2025.1610190

**Published:** 2025-07-11

**Authors:** Yoshihito Kitamura, Seiichi Shimizu, Shintaro Kuroda, Naruhiko Honmyo, Ryosuke Nakano, Hiroshi Sakai, Hiroyuki Tahara, Masahiro Ohira, Kentaro Ide, Tsuyoshi Kobayashi, Tatsuo Ichinohe, Hideki Ohdan

**Affiliations:** ^1^ Department of Gastroenterological and Transplant Surgery, School of Biomedical and Health Sciences Hiroshima University, Hiroshima, Japan; ^2^ Department of Hematology and Oncology, Research Institute for Radiation Biology and Medicine, Hiroshima University, Hiroshima, Japan

**Keywords:** agranulocytosis, durvalumab, immune-related adverse event, intrahepatic cholangiocarcinoma, conversion surgery

## Abstract

We report a rare hematological immune-related adverse event (irAE) induced by durvalumab, an immune checkpoint inhibitor, after conversion surgery in a patient with intrahepatic cholangiocarcinoma (ICC). A 72-year-old male was admitted for recurrent ICC treatment in the posterior sector. The primary treatment chosen was systemic chemotherapy with durvalumab combined with gemcitabine and cisplatin. After eight cycles of chemotherapy, partial hepatectomy was performed against three nodules of ICC at liver segments 6 and 7 as conversion surgery. Approximately 3 months after the last injection of durvalumab, the patient was readmitted to our department due to a high fever. The number of neutrophils dropped to zero. Despite daily administration of granulocyte colony-stimulating factor, the patient had agranulocytosis with neutrophil counts remaining between 0 and 80/µL for 16 days. The results of the bone marrow biopsy indicated that the patient had cytotoxicity due to autoimmunity against the common progenitor cells of granulocytes and monocytes as an adverse reaction to durvalumab. The patient was diagnosed with immune-related neutropenia and started on steroid bolus therapy. Four days after initiating steroid therapy, the neutrophil count began to improve, reaching 1540/µL after 7 days, and remained stable thereafter. This case highlights the potential for durvalumab-induced immune-related agranulocytosis following conversion surgery in a patient with ICC, emphasizing the importance of careful monitoring and timely management of irAEs, particularly in the context of postoperative infections.

## 1 Introduction

Biliary tract cancers, including intrahepatic cholangiocarcinoma (ICC), have a poor prognosis because they are usually diagnosed at an advanced stage and definitive surgery is difficult to perform (1). To overcome this limitation in curative therapy, the treatment strategy consists of the combination of surgery, chemotherapy, and immune checkpoint inhibitors (ICIs).

ICIs, programmed cell death ligand 1 (PD-L1), and cytokine T-lymphocyte–associated protein 4 (CTLA-4) inhibitors, are drugs that enhance the immune response against tumors by binding to inhibitory receptors or their ligands, which are immune checkpoint molecules, and activating T cells (2). The addition of immunotherapy to chemotherapy has demonstrated improved outcomes compared to chemotherapy alone in multiple solid tumor types. A recent landmark study, the phase 3 TOPAZ-1 trial, showed that durvalumab plus gemcitabine and cisplatin significantly improved overall survival of patients with advanced biliary tract cancer (3).

Immune checkpoint molecular signaling is important for the homeostasis of an organism, preventing autoimmune tolerance and excessive immune activation. Therefore, ICIs have been reported to cause autoimmune and inflammatory disease-like side effects, despite their important clinical benefits. However, there is insufficient knowledge regarding the variety, severity, and timing of ICI-related adverse events.

Here, we report a case of immune-related agranulocytosis that occurred 3 months after the last administration of durvalumab.

## 2 Case description

**Figure 1:**
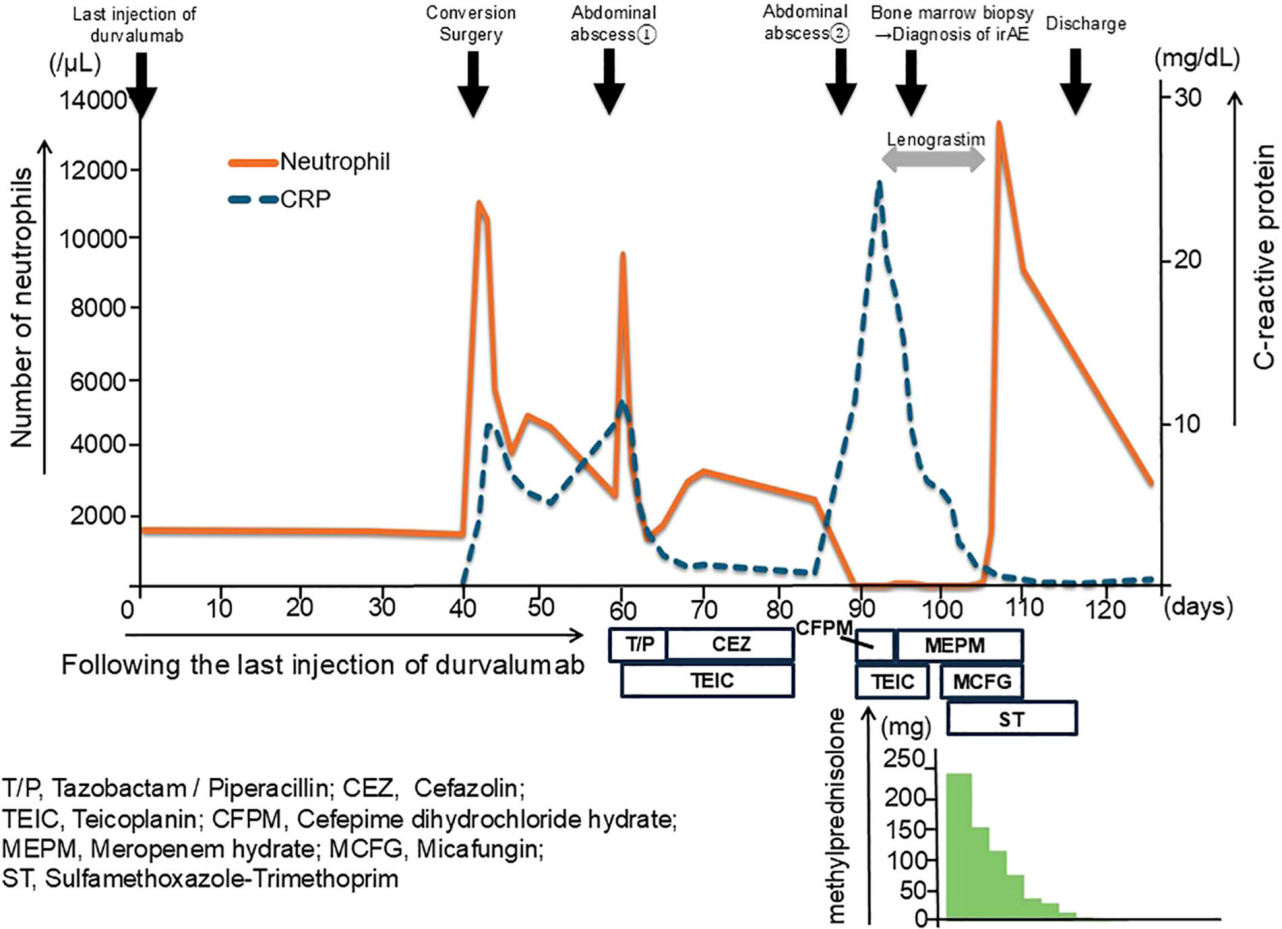
Clinical course and neutrophil count following durvalumab administration and conversion surgery. The clinical course of the patient over time, highlighting the key events and corresponding neutrophil counts. After 40 days from the last injection of durvalumab (day 0), the patient underwent conversion surgery (day 40). A bone marrow biopsy was performed to investigate persistent neutropenia. The patient was treated with lenograstim to stimulate neutrophil production, followed by high-dose methylprednisolone (mPSL) therapy starting at 250 mg/day that was tapered over time. Neutrophil counts significantly improved following steroid therapy initiation, with counts stabilizing as the dose was gradually reduced.

**Figure 2:**
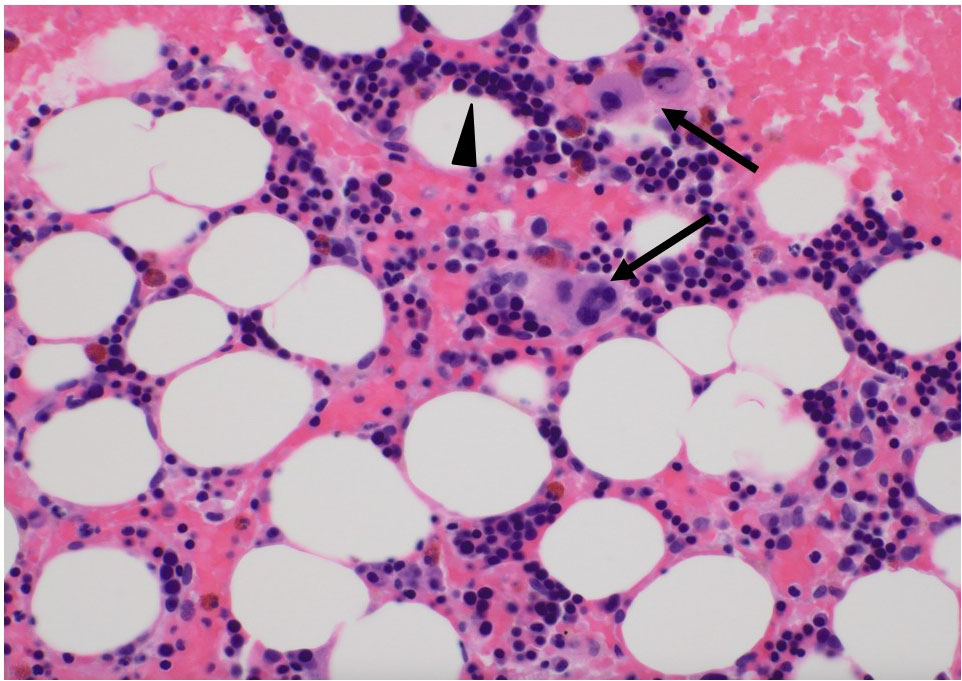
Pathological findings of bone marrow biopsy. Bone marrow biopsy revealed that myeloid progenitor cells were barely present; however, erythroblast and megakaryocyte counts were within normal limits. The arrows depict megakaryocytes, and arrow heads show erythroblasts. Most of the mononuclear cells were erythroblasts.

A 72-year-old male patient was admitted to our department for treatment of recurrent ICC in the posterior sector. He had a history of ischemic heart disease and diabetes mellitus, as well as anterior sectionectomy and partial hepatectomy at segment 6 due to primary ICC 3 years prior. After liver resection, the patient received adjuvant chemotherapy with oral S-1 plus gemcitabine. During follow-up for 2 years, there was no evidence of recurrent ICC. Enhanced CT and EOB-MRI revealed three tumors, initiating suspicion of ICC recurrence. Systemic chemotherapy with durvalumab, combined with gemcitabine and cisplatin, was selected as the primary treatment for recurrent ICC. The patient did not undergo comprehensive genomic profiling or a gene panel test during treatment. Durvalumab in combination with gemcitabine and cisplatin was administered intravenously in 21-day cycles. The standard regimen consisted of durvalumab (1500 mg, day 1), gemcitabine (1000 mg/m², days 1 and 8), and cisplatin (25 mg/m², days 1 and 8). Given the patient’s advanced age, treatment was initiated at reduced doses: gemcitabine (800 mg/m²), cisplatin (20 mg/m²), and durvalumab (1500 mg) for the first four cycles. Due to the development of neutropenia, cisplatin was discontinued and the gemcitabine dose was subsequently reduced to 700 mg/m² from the fifth cycle onward (**Supplementary Figure 1**). After the administration of eight cycles of chemotherapy, its effect was assessed as having resulted in stable disease according to the Response Evaluation Criteria in Solid Tumors (RECIST) version 1.1. Based on this finding, partial hepatectomy was performed for three ICC nodules in liver segments 6 and 7 as conversion surgery. Pathological findings showed moderate-to-poorly differentiated ICC, although there was no evidence of necrotic changes in the resected specimens. Postoperative CT tomography revealed no residual tumors in the remnant liver. The patient was discharged on postoperative day 13 without complications. Twenty days after the conversion surgery, the patient developed an abdominal abscess. Treatment was initiated with tazobactam/piperacillin, and teicoplanin was subsequently added based on blood culture results that were positive for gram-positive cocci. Final culture results from the abscess and blood revealed Staphylococcus lugdunensis and Propionibacterium species. Based on the susceptibility profile, the patient was treated with cefazolin and teicoplanin along with drainage of the abscess, resulting in clinical improvement. Notably, neutropenia was not observed during treatment of this initial infection. Eighty-nine days after the last administration of durvalumab, the patient was readmitted with high fever. Laboratory tests at the time of readmission revealed a neutrophil count of zero. Initially, agranulocytosis was suspected to be associated with severe infection arising from a recurrent abdominal abscess. Percutaneous transhepatic drainage was performed, and empiric therapy with cefepime and teicoplanin was initiated based on the prior susceptibility results, along with granulocyte colony-stimulating factor (G-CSF). The culture results of the recurrent abscess again identified the presence of Propionibacterium species, and antibiotic therapy was changed to meropenem in accordance with the updated susceptibility data. Despite daily administration of G-CSF and broad-spectrum antibiotics, the patient remained agranulocytic, with neutrophil counts ranging from 0 to 80/µL for 16 days (**Figure 1**). Antinuclear antibodies were negative, and anti-granulocyte antibodies were not assessed. Both serum cortisol and thyroid hormone levels were within normal limits. A bone marrow biopsy performed by a hematologist revealed that myeloid progenitor cells were rarely observed, and erythroblast and megakaryocyte counts were within normal limits (**Figure 2**). Based on these findings, the patient was diagnosed with immune-related agranulocytosis, possibly caused by autoimmunity against common progenitor cells of granulocytes and monocytes, as an adverse effect of durvalumab. Steroid therapy at a dose of 250 mg/day (5 mg/kg/day) was administered for 3 days because of immune-mediated neutropenia, and filgrastim was administered to accelerate neutrophil recovery. The steroid dose was tapered by half every 2 days. Neutrophils in peripheral blood were detected 4 days after starting steroid bolus therapy at an absolute number of 10/µL. This count increased to 1540/µL after 7 days of steroid treatment, and remained stable during subsequent checks (**Figure 1**). The patient was discharged without any signs of infection or adverse events. Thirteen months have since passed following the treatment for severe neutropenia, and there has been no recurrence of neutropenia or any other irAEs.

## 3 Discussion

To improve the poor prognosis of biliary tract cancers, a treatment and management algorithm for locoregional and advanced or metastatic disease is provided by various societies or congresses, including the National Comprehensive Cancer Network and European Society for medical oncology (4, 5). The phase III TOPAZ-1 trial demonstrated that durvalumab combined with chemotherapy significantly improved overall survival and progression-free survival compared to placebo plus chemotherapy. Based on these results, durvalumab in combination with gemcitabine and cisplatin is also a recommended treatment option for patients who develop recurrent disease more than 6 months after surgery with curative intent and more than 6 months after completion of adjuvant therapy (4).

Although ICIs represent a breakthrough in cancer treatment, they present a clinical dilemma regarding adverse events. The reported irAEs are heterogeneous and can occur in various organs, including the skin, gastrointestinal, endocrine, and respiratory systems. Regarding the frequency of irAEs, the TOPAZ-1 trial revealed a rate of 12.7%, with grade 3 or 4 irAEs occurring in 2.4% of patients in the durvalumab group. Although no hematological irAEs were evident in this study, any hematological adverse events could be included as treatment-related adverse events (6). Regarding hematological irAEs, a review showed a frequency of 3.6% for all grades and 0.7% for grade 3 or 4. The review reported that neutropenia was profound and severe, with neutrophil counts close to 0/mm^3^ in most of the cases (7). In our case, the patient experienced severe neutropenia with neutrophil counts nearing 0/mm³, which persisted for 16 days despite GCSF administration. Prophylactic antifungal therapy was administered along with antibiotics to prevent complications from severe infections.

Recently, reports of immune-related agranulocytosis have been increasing, leading to a better understanding of this condition. The median time from the initiation of ICIs to the onset of agranulocytosis has been reported to be approximately 10–11 weeks, while some studies involving combination ICI therapy have shown a shorter interval of approximately 6.4 weeks. Cases of delayed immune-related agranulocytosis have also been reported, with one study describing onset at 92 days after ICI administration (8). Immune-related agranulocytosis is typically diagnosed through differential blood counts. Additional diagnostic tools may include bone marrow aspiration, autoantibody testing, cytogenetic analysis, and bone marrow biopsy. Histological findings in bone marrow biopsy vary widely, ranging from normocellular marrow to granulocytic maturation arrest or even complete absence of myelopoiesis. To exclude other causes of agranulocytosis, several reports have incorporated viral testing (including CMV, EBV, and Parvovirus B19), evaluations for autoimmune diseases (e.g., rheumatologic markers and endocrine profiles), and assessments for hematologic malignancies (e.g., gene panels or flow cytometry) (9, 10). In our case, although a comprehensive viral and autoimmune workup was not performed, the clinical course and bone marrow biopsy findings were consistent with a diagnosis of immune-related agranulocytosis. While no standardized treatment for this condition exists, prior reports suggest that immunosuppressive therapy with systemic corticosteroids or non-steroidal agents (such as cyclosporine), in combination with G-CSF to promote neutrophil recovery and broad-spectrum antibiotics for infection control, may be effective. Most patients with Grade 4 neutropenia have been reported to respond to G-CSF therapy, with neutrophil recovery being achieved in approximately 90% of cases (10).

The mechanisms of pharmaceutical-induced agranulocytosis can be divided into immunological mechanisms, in which the drug causes the production of anti-neutrophil antibodies, and toxic mechanisms, in which the drug or its metabolites directly injure the progenitor cells of the granulocyte system in the bone marrow (11). Antinuclear or antigranular antibody assays may be helpful to diagnose the autoimmune nature of neutropenia. Although the presence of anti-granulocyte antibodies was not measured, it was presumed that antibody-dependent cellular cytotoxicity was triggered specifically in the granulocyte lineage, as granulocyte progenitor lineage cells were difficult to detect in bone marrow specimens.

In recent years, there has been an increase in reports of conversion surgery being performed after preoperative treatment using ICIs for certain tumors, including esophageal, lung, and gastric cancers (12–14). When we reviewed hepatectomy cases treated with ICIs before surgery, the atezolizumab plus bevacizumab regimen had a remarkable ability in downstaging tumors in unresectable HCC, facilitating treatment conversion and promoting cancer-free status (15, 16). Although the durvalumab plus tremelimumab regimen is also a viable option for patients with unresectable HCC and showed a promising survival rate of 30.7% at 3 years, the rate of conversion surgery has not been reported in any of the papers (17).

When conversion surgery becomes feasible after chemotherapy, the timing of irAEs should be carefully considered while establishing treatment strategies. The timing of irAEs typically ranges from a few weeks to a few months after ICI administration (7). The general frequency of irAEs is reported to be 20–30% (18), and the risk of irAEs must be carefully weighed against the clinical benefits of ICI therapy when determining the optimal timing for surgery. Previous studies have shown a correlation between irAE occurrence and improved outcomes such as objective response rate, progression-free survival, and overall survival (19). Patients eligible for conversion surgery are those who have demonstrated antitumor efficacy from ICI therapy, such as tumor shrinkage or the absence of new lesions; however, these patients are also considered to be at a higher risk of developing irAEs.

The occurrence of irAEs in relation to surgery and postsurgical complications must be carefully considered. In this case, neither the surgery itself nor the initial abdominal abscess led to the onset of irAEs, as the patient’s neutrophil count increased following these events. However, in the second episode of the abdominal abscess, the patient’s neutrophil count dropped rapidly, and a bone marrow biopsy revealed the disappearance of granulocyte progenitor cells. Autoimmune diseases such as systemic lupus erythematosus are generally known to cause cytopenia, including neutropenia (20). These diseases can worsen because of the physical stress caused by infection or other factors. In this case, we postulated that the development of an abdominal abscess triggered an irAE, which in turn caused agranulocytosis. The exact mechanism linking postsurgical infections to the development of irAEs remains unclear. However, this case underscores the potential role of postsurgical infections as a trigger for irAEs, highlighting the need for vigilant postoperative management, particularly in the context of persistent or refractory infections. Therefore, close monitoring of irAEs should be an integral part of the care plan for patients undergoing conversion surgery after ICI therapy.

Although conversion surgery can be delayed for several months after chemotherapy to reduce the risk of irAEs, performing surgery at an oncologically optimal time is often preferable. Therefore, the timing of surgery must balance the benefits of resection with the risks of irAEs, particularly in patients who respond well to ICI therapy.

This case highlights the development of immune-related agranulocytosis as a rare hematological adverse event following durvalumab treatment in a patient with ICC. This suggests that postoperative infections may trigger irAEs in such patients. As conversion surgery becomes more common following ICI therapy, further studies are needed to clarify the risk factors for irAEs and develop strategies for safely managing these patients. Accumulating evidence from larger cohorts will help better understand how to balance the therapeutic benefits of ICIs with the risks of potentially life-threatening irAEs, especially in the context of conversion surgery.

## 4 Conclusion

Herein, we reported a rare hematological irAE induced by durvalumab after conversion surgery in a patient with ICC. Because the standard chemotherapy regimen for ICC includes ICI therapy, the possibility of irAEs, including severe neutropenia, should be closely monitored to ensure timely intervention and management.

## Data Availability

The original contributions presented in the study are included in the article/**Supplementary Material**. Further inquiries can be directed to the corresponding author.

## Supplementary material

Supplementary Figure 1 Chemotherapy schedule before conversion surgery. Durvalumab with gemcitabine and cisplatin was administered intravenously on a 21-day cycle. Considering the age of the patient, he was started on gemcitabine 800 mg/m^2^, cisplatin 20 mg/m^2^, and durvalumab 1500 mg for four cycle. Following completion of this regimen, gemcitabine was reduced to 700 mg/m^2^ and cisplatin was discontinued due to neutropenia.
