# Supplementary figures and images for: Durvalumab induced immune-related agranulocytosis after conversion surgery in a patient with intrahepatic cholangiocarcinoma: a case report
Source: Front Immunol. 2025 Jul 11;16:1610190. doi: 10.3389/fimmu.2025.1610190 (PMC12289667; doi:10.3389/fimmu.2025.1610190)

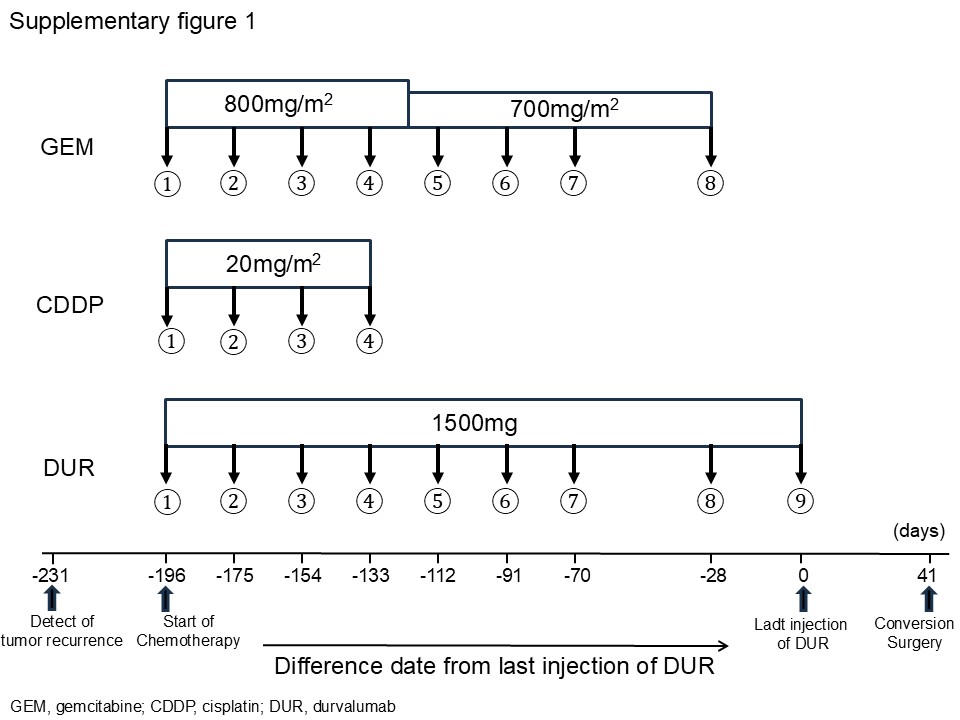

Supplement: Supplementary Figure 1 — Chemotherapy schedule before conversion surgery. Durvalumab with gemcitabine and cisplatin was administered intravenously on a 21-day cycle. Considering the age of the patient, he was started on gemcitabine 800 mg/m2, cisplatin 20 mg/m2, and durvalumab 1500 mg for four cycle. Following completion of this regimen, gemcitabine was reduced to 700 mg/m2 and cisplatin was discontinued due to neutropenia. [file Image1.jpeg]
